# Histological damage and inflammatory response elicited by *Monobothrium wageneri *(Cestoda) in the intestine of *Tinca tinca *(Cyprinidae)

**DOI:** 10.1186/1756-3305-4-225

**Published:** 2011-12-07

**Authors:** Bahram Sayyaf Dezfuli, Luisa Giari, Samantha Squerzanti, Alice Lui, Massimo Lorenzoni, Sidika Sakalli, Andrew P Shinn

**Affiliations:** 1 Department of Biology & Evolution, University of Ferrara, St. Borsari 46, 44123 Ferrara, Italy; 2 Department of Cellular and Environmental Biology, University of Perugia, St. Elce di Sotto 5, 06123 Perugia, Italy; 3 Institute of Aquaculture, University of Stirling, Stirling FK9 4LA, Scotland, UK

**Keywords:** Caryophyllidean, tapeworm, mucous cells, granulocytes, immune response

## Abstract

**Background:**

Among the European cyprinids, tench, *Tinca tinca *(L.), and the pathological effects their cestodes may effect, have received very little or no attention. Most literature relating to *Monobothrium wageneri *Nybelin, 1922, a common intestinal cestode of tench, for example, has focused on aspects of its morphology rather than on aspects of the host-parasite interaction.

**Results:**

Immunopathological and ultrastructural studies were conducted on the intestines of 28 tench, collected from Lake Piediluco, of which 16 specimens harboured tight clusters of numerous *M. wageneri *attached to the intestinal wall. The infection was associated with the degeneration of the mucosal layer and the formation of raised inflammatory swelling surrounding the worms. At the site of infection, the number of granulocytes in the intestine of *T. tinca *was significantly higher than the number determined 1 cm away from the site of infection or the number found in uninfected fish. Using transmission electron microscopy, mast cells and neutrophils were frequently observed in close proximity to, and inside, the intestinal capillaries; often these cells were in contact with the cestode tegument. At the host-parasite interface, no secretion from the parasite's tegument was observed. Intense degranulation of the mast cells was seen within the *submucosa *and *lamina muscularis*, most noticeably at sites close to the tegument of the scolex. In some instances, rodlet cells were encountered in the *submucosa*. In histological sections, hyperplasia of the mucous cells, notably those giving an alcian blue positive reaction, were evident in the intestinal tissues close to the swelling surrounding the worms. Enhanced mucus secretion was recorded in the intestines of infected tench.

**Conclusions:**

The pathological changes and the inflammatory cellular response induced by the caryophyllidean monozoic tapeworm *M. wageneri *within the intestinal tract of an Italian population of wild tench is reported for the first time.

## Background

*Monobothrium wageneri *Nybelin, 1922 was originally described from specimens collected from wild *Tinca tinca *(L.) caught in northern Italy during the nineteen century. Since this first report, this tapeworm has been subsequently reported in Poland, Bohemia and the United Kingdom [1]. Several species of caryophyllidean cestodes are recorded from tench populations across Continental Europe, but only *M. wageneri *is reported to be specific to this host [1]. Among the European cyprinids, tench and the pathological effects their cestodes may effect, have received very little or no attention. Most literary accounts of *M. wageneri *appear to focus on their morphology rather than their impacts on their host [2].

The alimentary tract represents the primary route of parasitic infection in fish and other vertebrates [3]. Protozoan or helminths exert their effects on intestinal tissue either through their adhesion to it or their penetration through it [4]. Parasitic infections can cause several changes in the immune response [5], frequently provoking an inflammatory response resulting in variable numbers and types of leucocytes subsequently being observed in the epithelium and *lamina propria *of host tissue [6-8].

Inflammation is a protective reaction initiated by the vascular and cellular tissues of the host in response to physical injury or disease, and the inflammatory response displayed by many fish species to parasitic infection has been comprehensively described [9]. Inflammation causes a series of chemical and morphological changes in affected tissues including leucocyte migration and the formation or increase in the number of granulocytes. Chronic inflammatory reactions are easy to observe by histological approaches in fish [9].

The innate immune system produces the primary response to pathogens and comprises: 1) cells that are either phagocytic (*i.e*. granulocytes, macrophages) or are cytotoxic (*i.e*. natural killer); 2) proteins that mediate the responses (*e.g*. complement) which initiates inflammation or cytokines that control the cellular components; and, 3) physical and chemical barriers (*e.g*. epithelial barriers and anti-microbial peptides) that the body uses to prevent or deter the penetration and/or colonisation of pathogens [10]. Two types of granulocytes, mast cells (MCs) and neutrophils, have been reported to play a critical role as part of the defence function against pathogens and evidence for their involvement in the immune system of fish is growing [11-15].

MCs, also known as eosinophilic granule cells [16], are normally found in the connective tissues of the tegumentary, urinary, gastrointestinal, respiratory and reproductive systems of some fish species and are reported from all vertebrate groups [17]. Given the cytochemical features and location of MCs in fish host tissues, it has been suggested that they are analogous, both in the structural and functional properties they possess, to mammalian MCs [16,18]. It is, however, now widely accepted that MCs have a role in the immune response of fish [12,17,19]. MCs are motile and their abundance and distribution changes in response to parasitic infection with cells migrating to the site of infection [13,19,20]. At the site of parasitic infection, the MCs release their contents which include various tryptases, antimicrobial peptides, lysozyme and piscidin [12,18,19,21]. How these MCs degranulate in response to the presence of parasites is detailed in the studies of Dezfuli *et al*. [19,21].

Mast cell secretions may have a role in attracting other types of cells (*i.e*. neutrophils), which are involved in the inflammatory process, especially during the period of initial pathogen challenge [22]. Neutrophils are one of the first cell types to arrive at the sites of inflammation and are a critical component of the teleost innate immune defence [23]. Neutrophils, like mast cells, are also phagocytic; their numbers increase in response to parasitic infection, ingesting foreign particles [24]. Neutrophils also migrate, accumulating at the site of parasitic infection or injury [6,24].

In addition to these cell types, rodlet cells (RCs), which are exclusive to fish and are commonly found in the epithelial tissue of all fish species, despite their structure being well established, their function and nature remains a subject of debate [25]. They are associated with the generalised host response stimulated by a variety of external stressors including parasitic infections [16,26] and there is now a consensus among most investigators that rodlet cells are endogenous cells [12]. The role of RCs as teleost inflammatory cells has been rarely studied in the past, although some data is available [13,26].

Until recently there had been little direct evidence of innate immune mechanism within the mucosal epithelium in response to parasitic infection in fish [15]. Fish mucus is involved in a wide range of functions, including feeding, excretion, reproduction, respiration, ionic and osmotic regulation, and in protection against, and resistance to, disease [27,28] and parasitic infection [29]. Mucous cells in some fish species, however, have been reported to produce and release defensive substances in response to infection [30].

To cope with its host's defence systems, adult cestodes possess "scolex gland cells" or "scolex glands" (*e.g*., [31]), the topic of which is discussed in the review of Whittington & Cribb [32]. The secretions produced from these glands have been reported to be histolytic [33] or to provide protection from the host's immune system [34]. The main aim of this paper is to detail the pathological changes induced by *M. wageneri *within the intestines of tench including observations on the ultrastructure of the host cellular responses to infection.

## Results

### Light microscopy

**Figure 1:**
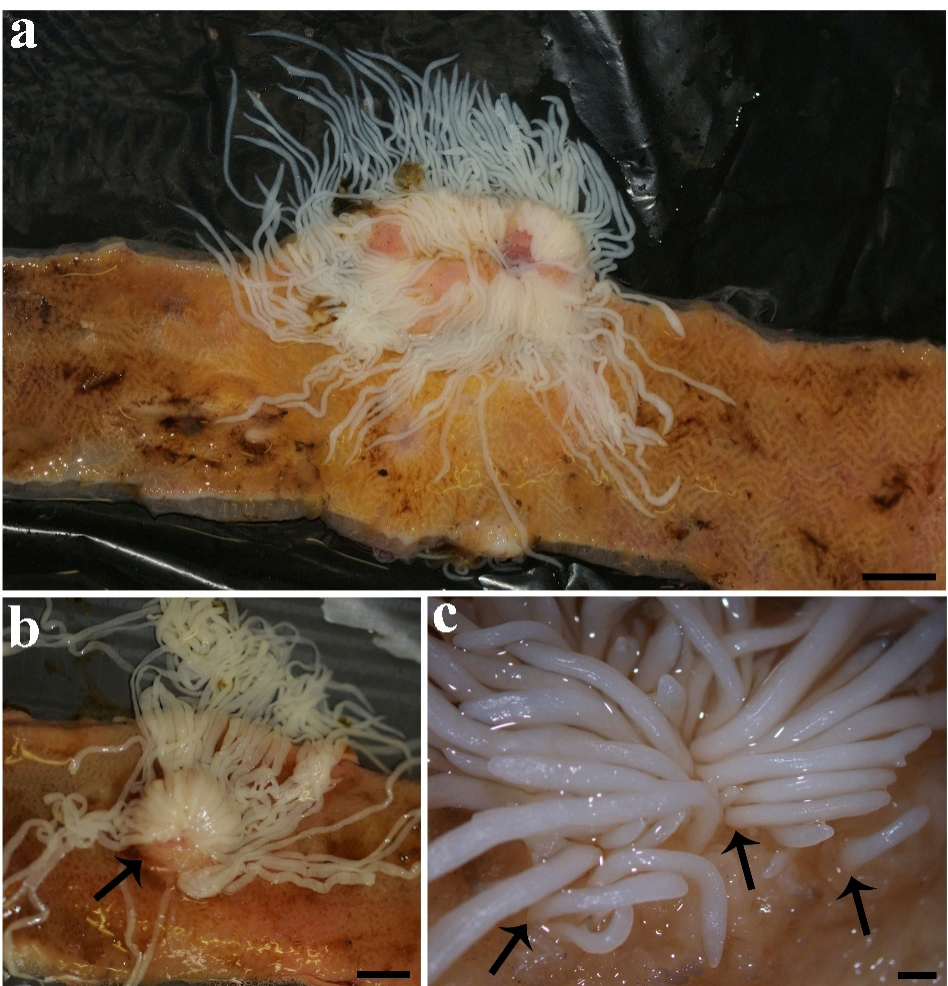
**A heavy infection of *Monobothrium wageneri***. **(a) **A heavy infection of *Monobothrium wageneri *comprising over 100 tapeworms in a single cluster, scale bar = 1 cm. **(b) **Attachment of *M. wageneri *resulted in the formation of a raised, rounded nodule (arrow) surrounding the worms, scale bar = 5 mm. **(c) **Anterior intestine of a tench, *Tinca tinca*, infected with *M. wageneri*; note the deep penetration (arrows) of the scolex and neck of worm, scale bar = 1.3 mm.

Sixteen (57.1%) of the 28 tench were infected with *M. wageneri*. The intensity of infection ranged from 1 to 103 worms per host (35.8 ± 41.5, mean ± standard deviation [S.D.]), with *M. wageneri *specimens encountered primarily within the anterior part of the intestine (Figure 1a). The attachment of the *M. wageneri *resulted in the formation of a raised, rounded nodule surrounding the tapeworms (Figure 1b). Tapeworms were commonly observed in tight clusters of a variable number (Figure 1c). The heaviest infection (103 worms) observed in one tench, were found in a single cluster of parasites within the host's anterior intestine (Figure 1a), although an infection of 100 worms found in a second tench consisted of 4 discrete foci. The clusters contained primarily adult parasites; juvenile tapeworms were rare and were found in only 2 hosts. These infections frequently caused swellings that were evident as raised, rounded nodules visible from the exterior of the intestine prior to dissection. The swelling induced by the attachment of the cestodes and their white colouration permitted their ready detection during *post mortem *examination.

**Figure 2:**
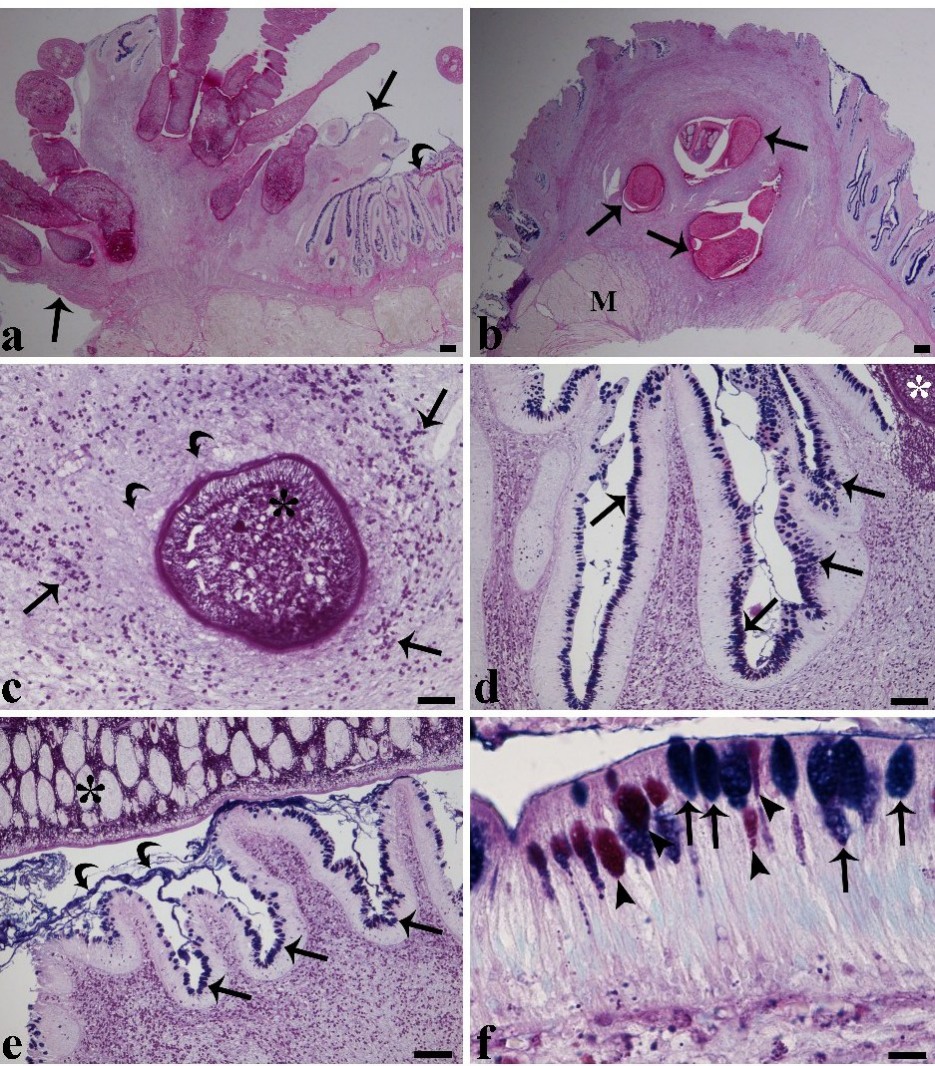
**Transverse section through the intestine of a tench infected with several *Monobothrium wageneri***. **(a) **Transverse section through the intestine of a tench infected with several *Monobothrium wageneri *showing a pronounced inflammatory response surrounding the scoleces and a marked lack of epithelia at the site of tapeworm attachment (arrows). Note the presence of intact epithelia (curved arrow) in close proximity to the nodule, scale bar = 200 μm. **(b) **Focal attachment of several *M. wageneri *penetrating the intestine of a tench as far as the *muscularis*. An intense host cellular reaction around the scoleces is visible (arrows), M = *muscularis*, scale bar = 200 μm. **(c) **Scolex of *M. wageneri *(asterisk) is surrounded by numerous granulocytes (arrow heads) and collagenous fibres (curved arrows), scale bar = 50 μm. **(d) **A high number of mucous cells (arrows) are visible within the epithelia in the immediate vicinity of the scolex (asterisk). Note the intense recruitment of granulocytes (arrow heads) within the *sub-mucosa*, scale bar = 100 μm. **(e) **The villi adjacent to the body of the cestode (asterisk) are seen to be covered with a blanket of mucus (curved arrows) and possess a high number of mucous cells (arrows). Numerous granulocytes (arrow heads) are evident within the *sub-mucosa*, scale bar = 100 μm. **(f) **Alcian blue/PAS stained mucous cells close to the site of cestode attachment. The arrows indicate that most mucous cells stain positively for acid glycoconjugates whilst the arrow heads indicate that a lower number of mucous cells stain for the presence of mixed glycoconjugates, scale bar = 10 μm.

Attachment involved penetration of the parasite's blunt, rounded scolex, deep into the intestine wall (Figure 2a). The scolex extended deep into the *mucosa *and *submucosa *as far as the *muscularis *layer (Figure 2b). The focal attachment of tapeworms, the lateral expansion of their scoleces, and the formation of a raised, rounded nodule within the intestine provided for the secure attachment of the cestodes to their host (Figure 2a).

Infections of adult *M. wageneri *invoked a pronounced, progressive fibrogranulomatous response that extended throughout all layers of the intestine (Figure 2b). This chronic host reaction was associated with a complete loss of normal gut architecture and the replacement of the *mucosa*, *submucosa *and *muscularis *layer with inflammatory tissue (Figures 2a, b) in both light and heavily parasitised tench. At the site of attachment, the cestodes caused necrosis and degeneration or loss of the epithelium (Figure 2a). Within the *submucosa *layer, beneath the point of scolex insertion, numerous granulocytes (*e.g*., MCs, neutrophils) (Figure 2c) and collagenous fibres were seen. The number of granulocytes in the infected intestines at the sites of parasite attachment (141 ± 51, mean ± S.D., n = 12) were significantly higher than those found in zones approximately 1 cm away from the point of attachment (74 ± 38, mean ± S.D., n = 12) and in the intestines of uninfected tench (76 ± 17, mean ± S.D., n = 12) (ANOVA, p < 0.01). There was, however, no significant difference between the number of granulocytes found in the latter two sets of samples (*i.e*. 1 cm from the point of cestode attachment and in uninfected tench; ANOVA, p > 0.05).

The presence of numerous mucous cells among the epithelial cells of the *M. wageneri *infected intestines, especially within the epithelia in close proximity to the cestode induced nodule were evident (Figures 2c, d). Parasitised intestines were determined to have a significantly higher number of mucous cells (40 ± 18, mean ± S.D., n = 12) than those that were uninfected (21 ± 17, mean ± S.D., n = 12) (ANOVA, p < 0.01). *In situ*, infected areas of intestine were covered by a yellowish catarrh which appeared as a thick, adherent blanket of mucus that gave an intense positive signal when stained with alcian blue (Figure 2e). These layers of catarrh covering the epithelium were most frequently observed in the intestines of infected fish in zones in close proximity (within 1 cm) to the site of *M. wageneri *attachment and adjacent to the body of a cestode (Figure 2e). The present study also investigated the occurrence of each type of mucous cell, using their reaction to alcian blue (AB) and periodic acid Schiff's (PAS) stains to categorise each type (Figure 2f). In infected intestines, AB positive mucous cells (24 ± 13, mean ± S.D., n = 12; Figure 2f) were significantly more numerous than those of uninfected fish (11 ± 8, mean ± S.D., n = 12) (ANOVA, p < 0.05). Similarly, AB/PAS positive mucous cells were found to be significantly higher in parasitised intestines (9 ± 7, mean ± S.D., n = 12) than in uninfected tench (4 ± 3, mean ± S.D., n = 12) (ANOVA, p < 0.01). There was, however, no significant difference in the number of PAS positive mucous cells between infected (7 ± 4, mean ± S.D., n = 12) and uninfected intestines (6 ± 7, mean ± S.D., n = 12) (ANOVA, p > 0.05). No discernible damage to the cestodes was observed.

### Transmission electron microscopy

**Figure 3:**
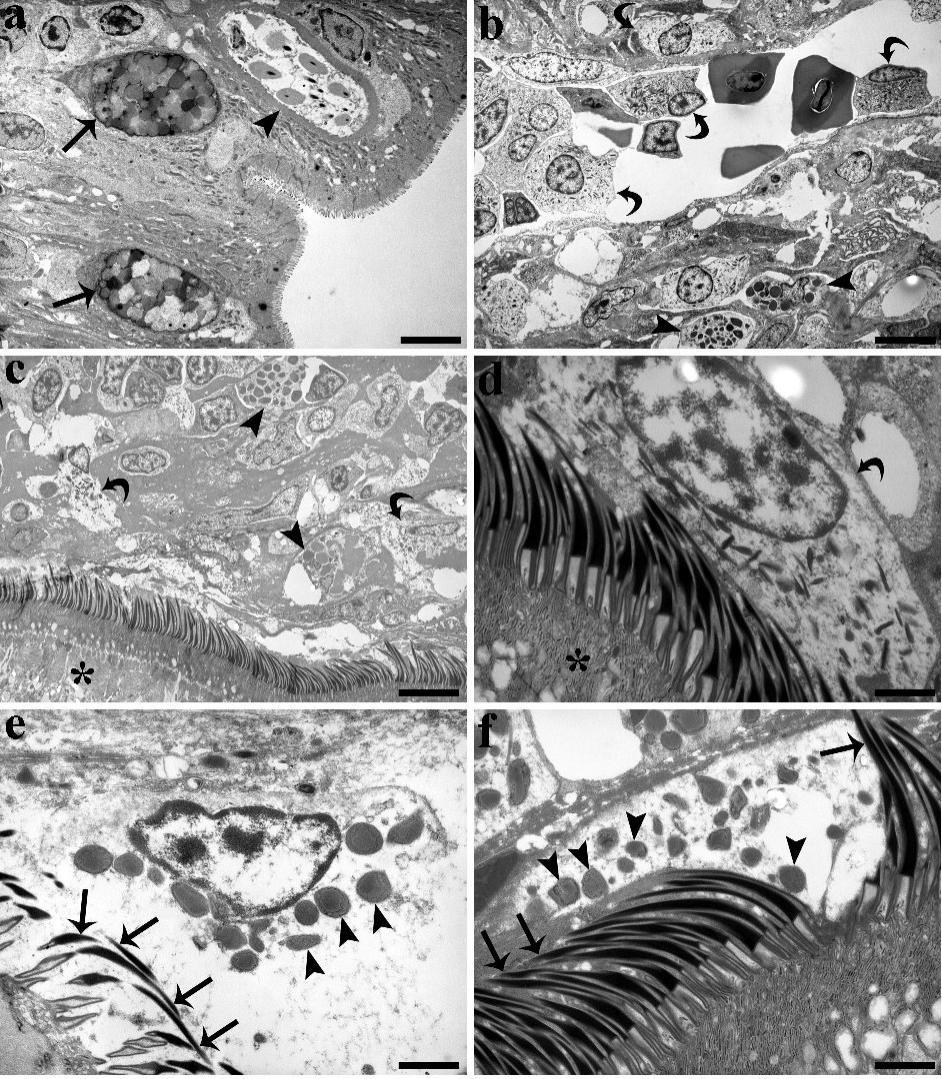
**A TEM section through two mucous cells (arrows) and a rodlet cell (arrow head) within the intestinal epithelium of a tench**. **(a) **A TEM section through two mucous cells (arrows) and a rodlet cell (arrow head) within the intestinal epithelium of a tench, *Tinca tinca*, infected with *Monobothrium wageneri*. Note that within the mucous cells there are electron-opaque and electron-lucent granules, scale bar = 4.3 μm. **(b) **Neutrophils (curved arrows) inside a capillary and within the connective tissue of the *sub-mucosa *of an infected tench. Arrow heads show the position of the mast cells within the connective tissue, scale bar = 4.4 μm. **(c) **Mast cells (arrow heads) and neutrophils (curved arrows) in close proximity to the scolex tegument of *M. wageneri *(asterisk), scale bar = 4.6 μm. **(d) **A neutrophil (curved arrow) attached to the scolex microtriches. Asterisk marks the scolex tegument, scale bar = 1 μm. **(e) **A mast cell releases its granules (arrow heads) in the vicinity of the scolex microtriches (arrows), scale bar = 0.5 μm. **(f) **Free mast cell granules (arrow heads) are visible among the scolex microtriches (arrows), scale bar = 1 μm.

In transmission electron microscopy (TEM) sections, the nucleus of the mucous cells was observed to be elongated and basally placed. Well developed rough endoplasmic reticulum, Golgi apparatus and a few round-elongated mitochondria were observed in the basal portion of the cell. Mucus granules occupy the entire supranuclear cytoplasm, appearing as spherules or polyhedrons surrounded by a single granule membrane. The mucus granules appeared electron-opaque and, in some instances, as electron-lucent (Figure 3a). RCs, in variable numbers (Figure 3a), were also observed among the epithelia of both *M. wageneri *infected and uninfected tench in zones in close proximity to and at a distance from the cestode induced nodules. In some of the infected intestines, MCs and neutrophils were also observed within the epithelia (not shown).

The inflammatory swellings surrounding the *M. wageneri *primarily consisted of fibroblasts, but also included a large number of two types of granulocyte: neutrophils and MCs. Interestingly, RCs were found to co-occur with these granulocytes within the *submucosa *of the nodule of infected intestines. Neutrophils and MCs were also recorded within the connective tissue surrounding capillaries and within the blood vessels within the *submucosa *and *muscularis *layer (Figure 3b). MCs were irregular in shape with an eccentric, polar nucleus, and a cytoplasm characterised by numerous large, electron-dense, membrane-bounded granules (Figure 3c). The cytoplasm typically contained two to three mitochondria and an inconspicuous Golgi apparatus. MCs were frequently surrounded by collagen fibres of the *submucosa *or by fibroblast-like unsheathing cells. Neutrophils were found to be numerous within the nodule, here they appeared round to oval in shape though their outline was commonly irregular. These cells also contained a round nucleus and a cytoplasm that contained dark, elongated granules which were fibrous in appearance (Figure 3d). Very few mitochondria and fragments of rough endoplasmic reticulum were observed in the cytoplasm.

Degranulation of the MCs, which was common in the *submucosa*, was visible by light microscopy. The degranulation of MCs was characterised by the conspicuous swelling of granules, with free granules frequently seen in close proximity to the capilliform filitriches (Figure 3e) or adjacent to/between the coniform spinitriches of the scolex (Figure 3f) (see [35] for cestode microtriche terminology). Neutrophils were seen in close contact with the microtriches of the scolex (Figure 3d). The MCs and neutrophils adjacent to the parasite tegument contained very few organelles and had a cytoplasm that appeared vacuolised, which were quite unlike the same cell types observed in zones further away from the body of the cestode.

## Discussion

Anthropochore fish movements are a common route of parasite introduction. As such, Kennedy [36] estimated that approximately 68.7% of all introduced parasites were through the movement of ornamental fish species into the country. In the same year, Gibson [1] suggested that the introduction and establishment of *M. wageneri *into Britain may have been through the importation of infected ornamental varieties of tench, for sport angling and fisheries, although he also discusses the possibility of parasite introduction via infected oligochaete worms, the suggested intermediate host, which are also imported from the Continent for bait. There are no known published records regarding the pathogenicity of *M. wageneri *in tench from Italy and the host cellular reaction and the results from the current study are among the first.

Seven species exist in the genus *Monobothrium *Diesing, 1863, although *M. wageneri *and *M. auriculatum *Kulakovskaya, 1961, which is recorded as infecting *Leuciscus danilewskyi *(Kesseler) in the Ukraine [2], are the only European representatives of this genus [37]. *Monobothrium ingens *Hunter, 1927 and *M. hunteri *Mackiewicz, 1963 recorded from the catostomidid cyprinids *Ictiobus cyprinellus *(Valenciennes) and *Catostomus commersoni *Lacépède from North America may exert pathogenic effects through the creation of lesions as a consequence of their attachment to the host's intestine [31,37,38]. The pathological changes induced by *M. wageneri*, however, differ markedly from those seen in other intestinal cyprinid-cestode systems (*e.g*. *Bothriocephalus acheilognathi *Yamaguti, 1934 and *Khawia sinensis *Hsü, 1935 infections in *Cyprinus carpio *L [39,40]). These differences include the magnitude of the inflammatory response, the involvement of all layers of the intestine, and the complete loss of gut architecture, even in tench with light tapeworm burdens. Pronounced inflammatory nodules, leading to the partial occlusion of the intestinal tract were found in hosts infected with as few as 6 adult *M. wageneri*. Although the focal attachment of *M. wageneri *limits the area of intestine damaged by this cestode, the tight clustering of tapeworms accentuates the severity of the individual lesions. Intestinal occlusion and rupture are unusual and are, according to Williams & Jones [41], extreme consequences of tapeworm infection. These are among the most serious impacts caused by intestinal tapeworms, which have been associated with nutritional disturbance, debilitation and even the death of heavily infected fish [42].

Inflammation of the intestinal tract can be provoked by a variety of factors including, for example, feed [43], infectious agents and chronic stress [44]. The level of infection and the tissue or organ affected can influence the range of histopathological responses initiated to an endoparasitic infection. These can range from benign encapsulation of the pathogen by host cells, to acute and chronic inflammation and necrosis [6]. The relative importance of the body organ that is infected and whether its function is unduly compromised, therefore, dictates host survival. Most pathology associated with cestode infections, however, results from the deep penetration of the scolex into the intestinal wall [45]. This is the case in *M. wageneri*, which induces marked pathological changes, penetrating the *muscularis *layer ([41] and current study), causing a significant inflammatory response in all layers of the intestine in both light and heavy infections.

Fish mucus is the intestine's first line of mucosal initiated defence [46]. Parasitic infections can induce hyperplasia and hypertrophy of mucous cells and can increase the level of mucus secretion in the intestine [29,47]. Recent investigations by Bosi *et al*. [47] and Dezfuli *et al*. [29] quantified the effects of enteric helminth infections on the density of mucous cells and on the composition of the mucus. Bosi *et al*. [47] determined the number of mucous cells in the intestines of uninfected brown trout, *S. trutta*, and in those infected with the acanthocephalan *Pomphorhynchus laevis *(Zoega in Müller, 1776) and found a significant difference in the number of cells between the two groups. Dezuli *et al*. [29], found a significant increase in the number of mucous cells staining positively for acid glycoconjugates in brown trout infected with both *Cyathocephalus truncatus *(Pallas, 1781) and *Echinorhynchus truttae *(Schrank, 1788). Data from the current study indicates that *M. wageneri *elicits a similar response with an increase in the number of two types of mucous cell - AB and AB/PAS positive cells. The study of Fairweather [48], likewise, documented different mucous cell secretions in response to the presence of parasites.

The attachment organs used by intestinal helminths during the process of attachment to their host's often induce inflammation in their host's alimentary tract [6-8]. RCs and two types of granulocytes, namely MCs [12,16] and neutrophils [11,14] have been repeatedly demonstrated to play a critical role in the immune system of fish as part of their defence function against pathogens. There is, therefore, a growing interest and accumulating body of evidence regarding the role of these inflammatory cells in the immune system of fish. Granulocytes are generally considered effector cells of the innate immune response [49]. Innate defence provides a pre-existing and fast-acting system of protection which is non-specific and has several advantages over the slow-acting specific immune responses [50]. The importance of each of these cells types *i.e*. RCs, MCs and neutrophils, will be discussed briefly in turn.

RCs occur in a wide range of tissues of teleosts and have been, most commonly, associated with epithelia [25]. The results of several recent investigations on both wild and farmed fish suggests that RCs represent an immune cell type closely linked to other piscine inflammatory cells [8,12,26]. In the present survey, however, the number of RCs in infected and uninfected tench were not determined because *M. wageneri *destroys the epithelia at the site of attachment. RCs were found to co-occur with MCs and neutrophils within the *submucosa *of cestode induced nodules. The findings of intestinal RCs at this site is unusual and requires further investigation.

Most teleosts possess MCs and these have been likened to mammalian mast cells in that they possesses similar structural and functional properties [16,18]. In fish infected with helminths, it has been observed that MCs tend to migrate and accumulate in large numbers at the site of infection [16,20]. In some fish-acanthocephalan systems, numerous MCs have been found at the sites of infection [8,51] while they have been notably lower in uninfected fish; a similar situation was seen in the current study in the tench infected with *M. wageneri*. Descriptive data exists detailing how MCs degranulate in response to their exposure to a variety of known degranulating agents and pathogens [18,19,52,53]. In parasitised tench, an intense MC degranulation was observed at the site of *M. wageneri *infection, notably in the immediate zone surrounding the scolex. It is possible that the secretions produced by the mast cells may have a role in attracting other cell types (*i.e*. neutrophils) involved in the inflammatory process, particularly during the period of initial pathogen challenge [17,22]. Murray *et al*. [52] suggested that MCs may be involved in the direct destruction of pathogens, adding to their multifunctional role in teleosts.

In the current study, a high number of neutrophils were found to co-occur with MCs within the *submucosa *at the attachment sites of *M. wageneri*. A similar finding was reported by Dezfuli *et al*. [13] investigating the livers of minnows, *Phoxinus phoxinus *(L.), infected with *Raphidascaris acus *(Bloch, 1779) larvae. Neutrophils are one of the first cell types to arrive at the sites of inflammation and are a critical component of the teleost innate immune defence system [23,54]. Neutrophils are present in high numbers in the blood and in hematopoietic organ pools as a reserve, and, under normal conditions, are rare in the tissues and body cavities [55]. Generally, macrophages co-occur with neutrophils to engulf extracellular pathogens into intracellular phagosomes and, through a series of events that lead to the maturation of the vacuole, destroy the invading agent in the newly "armed" phagolysosome [54]. Macrophages from the head kidney of rainbow trout, *Oncorhynchus mykiss *(Walbaum), were reported by Whyte *et al*. [56] to kill the larvae of the eye fluke *Diplostomum spathaceum *(Rudolphi, 1819), although this was dependent on the ratio of cells to parasites. Interestingly, no macrophages were found at the sites of *M. wageneri *attachment in the current study and yet the data gathered does not permit a definitive explanation for this lack of macrophages.

Neutrophils and macrophages might co-occur when the infecting agent is small and can be readily engulfed [50] or is of a small size (*e.g*., diplostomules of *D. spathaceum*) that it can be killed by host macrophages (see [56]). *Monobothrium wageneri*, however, is a tapeworm measuring several centimetres in length and so it is improbable that the scolex or the entire worm can be engulfed by host macrophages. The findings from the current study suggest that, *M. wageneri *may preferentially induce the recruitment of neutrophils and MCs.

Hayunga [31] draws attention to the important detail that despite the poorly developed mechanical apparatus of caryophyllideans, they do possess numerous frontal glands. Histochemical and experimental examinations suggest that secretions from the tegumentary glands of caryophyllidean tapeworms consist of neutral glycoproteins that may assist in protecting the worm from the host's immune response [34]. In the absence of definitive studies, the function of these glandular elements remain speculative [32]. From the TEM observations that were made within the current study, several glandular cytons within the syncytial tegument along the anterior and lateral parts of the *M. wageneri *scolex were observed (not shown). No discharge from these glands or the presence of an adhesive layer between the host and the cestode was evident. The occurrence of abundant immune cells at the site of attachment and in close contact with the scolex of *M. wageneri *perhaps dismisses the possibility that the secretions produced by the scolex tegumentary glands are responsible for distancing the cellular responses of infected *T. tinca*.

## Materials and methods

Twenty-eight tench, *T. tinca*, from Lake Piediluco (Province of Terni, Central Italy 42° 31' 01" N; 12° 45' 00" E) were caught by professional fishermen belonging to Piediluco Fish Consortium using a gill net that was deployed on several occasions over a period of 12 months (May to October 2010, April 2011). Fish were transferred alive to the Consortium's facility where the tench were subsequently euthanised using 125 mg L^-1 ^MS222 (tricaine methanesulfonate, Sandoz, Basel, Switzerland), their spinal cords were severed. Fish were then lengthed, 35.7 ± 4.5 cm (mean total length ± S.D.) and weighed, 1379 ± 557 g (mean weight ± S.D.). Fish were dissected and sexed before the digestive tract from each fish was removed and opened longitudinally and examined for helminths. For helminths found still attached to the gut, their position was recorded before a 15 × 15 mm piece of tissue surrounding the site of attachment was excised and then fixed in either chilled (4°C) bouin's or in 10% neutral buffered formalin for 24 h. The bouin fixed material was subsequently rinsed in several changes of 4°C 70% ethanol before being stored in the same medium until processed for histology. After fixation, the tissues were dehydrated through an alcohol series and then paraffin wax embedded using a Shandon Citadel 2000 Tissue Processor. After blocking out, 5 μm thick sections were cut and then stained with haematoxylin and eosin and/or alcian blue 8 GX pH 2.5 and periodic acid Schiff's reagent (AB/PAS). Several histological sections from each tissue block were examined and photographed using a Nikon Microscope ECLIPSE 80i. For TEM, 7 × 7 mm infected pieces of gut tissue were fixed in chilled 2.5% glutaraldehyde in 0.1 M sodium cacodylate buffer for 3 h. The fixed tissues were then post-fixed in 1% osmium tetroxide for 2 h and then rinsed and stored in 0.1 M sodium cacodylate buffer containing 6% sucrose for 12 h. Thereafter, the pieces of tissue were dehydrated through a graded acetone series and embedded in epoxy resin (Durcupan ACM, Fluka). Semi thin sections (1.5 μm) were cut on a Reichert Om U 2 ultra microtome and stained with toluidine blue. Ultra-thin sections (90 nm) were stained with 4% uranyl acetate solution in 50% ethanol and Reynold's lead citrate and then examined using an Hitachi H-800 transmission electron microscope. For each method, several uninfected pieces of corresponding intestine were also processed so that a direct comparison with the infected material could be made.

The numbers of granulocytes and mucous cells containing acid glycoconjugates (positive to alcian blue AB), neutral mucosubstances (PAS positive) and mixed glycoconjugates (AB/PAS positive) were counted in twelve tench infected with *M. wageneri *and in twelve uninfected tench. For each fish, two intestinal sections from two different tissue blocks were evaluated using a Nikon Microscope ECLIPSE 80i and computerised image analysis software (Nis Elements AR 3.0). For comparative purposes, the number of granulocytes in an area measuring 30,000 μm^2 ^was determined, using light microscopy, in 20 separate zones on each section (10 zones within the *submucosa *layer in close proximity to the sites of cestode attachment and in 10 randomly selected zones at a distance of 1 cm from the site of attachment). In addition, the number of mucous cells in 10 separate areas of intestinal epithelium each measuring 15,000 μm^2 ^were counted on each section taken from both infected (close to the site of parasite attachment) and uninfected tench.

Prior to analysis, the gaussian distributions (*i.e*. normality) and the homogeneity of variances of the data were assessed; the mucous cells data were square root-transformed to meet these assumptions. Using the software package Statistica 7, ANOVA repeated measures were performed to detect significant differences in the number of granulocytes and mucous cells determined from the uninfected and infected fish. A Bonferroni *post-hoc *test and a P < 0.05 level of significance were used throughout.

## Conclusions

*Monobothrium wageneri *infections in the intestines of tench inflict severe mechanical damage due to the tight clustering of worms, and the deep penetration of their scolices. The occurrence of this tapeworm induces an intense inflammatory response, which results in the migration and recruitment of mast cells and neutrophils to the site of infection.

## List of abbreviations

AB: alcian blue; MCs: mast cells; PAS: periodic acid Schiff; RCs: rodlet cells; SD: standard deviation; TEM: transmission electron microscopy.

## Competing interests

The authors declare that they have no competing interests.

## Authors' contributions

BSD performed field work, supervised the laboratory work and wrote the initial draft. LG, SS, AL and SS collected data, performed field and laboratory work and analysed data. ML collected data and performed field work. APS intellectually supported the study and corrected the manuscript drafts. All authors read and approved the final manuscript.
